# Suffix sorting via matching statistics

**DOI:** 10.1186/s13015-023-00245-z

**Published:** 2024-03-12

**Authors:** Zsuzsanna Lipták, Francesco Masillo, Simon J. Puglisi

**Affiliations:** 1 Department of Computer Science, University of Verona, Verona, Italy; 2 Helsinki Institute for Information Technology (HIIT), Helsinki, Finland; 3 Department of Computer Science, University of Helsinki, Helsinki, Finland

**Keywords:** Generalized suffix array, Matching statistics, String collections, Compressed representation, Data structures, Efficient algorithms

## Abstract

We introduce a new algorithm for constructing the generalized suffix array of a collection of highly similar strings. As a first step, we construct a compressed representation of the matching statistics of the collection with respect to a reference string. We then use this data structure to distribute suffixes into a partial order, and subsequently to speed up suffix comparisons to complete the generalized suffix array. Our experimental evidence with a prototype implementation (a tool we call sacamats) shows that on string collections with highly similar strings we can construct the suffix array in time competitive with or faster than the fastest available methods. Along the way, we describe a heuristic for fast computation of the matching statistics of two strings, which may be of independent interest.

## Introduction

Suffix sorting—the process of ordering all the suffixes of a string into lexicographical order—is the key step in construction of suffix arrays and the Burrows–Wheeler Transform, two of the most important structures in text indexing and biological sequence analysis [1–3]. As such, algorithms for efficient suffix sorting have been the focus of intense research since the early 1990s [4, 5].

With the rise of pangenomics [6, 7], there is an increased demand for indexes that support fast pattern matching over collections of genomes of individuals of the same species (see, e.g., [8–12]). With pangenomic collections constantly growing and changing, construction of these indexes—and in particular suffix sorting—is a computational bottleneck in many bioinformatics pipelines. While traditional and well-established suffix sorting tools such as divsufsort [13, 14] and sais [15, 16] can be applied to these collections, specialised algorithms for collections of similar sequences, perhaps most notably the so-called BigBWT program [17], are beginning to emerge.

In this paper we describe a suffix sorting algorithm specifically targeted to collections of highly similar genomes that makes use of the *matching statistics*, a data structure due to Chang and Lawler, originally used in the context of approximate pattern matching [18]. The core device in our suffix sorting algorithm is a novel compressed representation of the matching statistics of every genome in the collection with respect to a designated reference genome, that allows determining the relative order of two arbitrary suffixes, from any of the genomes, efficiently. We use this data structure to drive a suffix sorting algorithm that has a small working set relative to the size of the whole collection, with the aim of increasing locality of memory reference. Experimental results with a prototype implementation show the new approach to be faster or competitive with state-of-the-art methods for suffix array construction, including those targeted at highly repetitive data. We also provide a fast, practical algorithm for matching statistics computation, which is of independent interest.

The remainder of this paper is structured as follows. The next section sets notation and defines basic concepts. In the “Compressed matching statistics” section we describe a compressed representation of the matching statistics and a fast algorithm for constructing it. The “Comparing two suffixes via the enhanced CMS” section then describes how to use the compressed matching statistics to determine the relative lexicographic order of two arbitrary suffixes of the collection. The “Putting it all together” section describes a complete suffix sorting algorithm. We touch on several implementation details in the “Implementation details” section, before describing experimental results in the “Experiments” section. Reflections and avenues for future work are then offered.

A preliminary version of this work appeared in [19].

## Basics

A string *T* over an ordered alphabet $\Sigma$, of size $|\Sigma | = \sigma$, is a finite sequence $T=T[1..n]$ of characters from $\Sigma$. We use the notation *T*[*i*] for the *i*th character of *T*, |*T*| for its length *n*, and *T*[*i*..*j*] for the substring $T[i]\cdots T[j]$; if $i>j$ then $T[i..j]=\varepsilon$, where $\varepsilon$ is the empty string. The substring (or factor) $T[i..]=T[i..n]$ is called the *i*th suffix, and $T[..i]=T[1..i]$ the *i*th prefix of *T*. We assume throughout that the last character of each string is a special character $\$$, not occurring elsewhere in *T*, which is set to be smaller than every character in $\Sigma .$

Given a string *T*, the *suffix array* $\textit{SA}$ is a permutation of the index set $\{1,\ldots ,n\}$ defined by: $\textit{SA}[i]=j$ if the *j*th suffix of *T* is the *i*th in lexicographic order among all suffixes of *T*. The *inverse suffix array* $\textit{ISA}$ is the inverse permutation of $\textit{SA}$. The *LCP*-*array* is given by: $\textit{LCP}[1]=0$, and for $i\ge 2$, $\textit{LCP}[i]$ is the length of the longest common prefix (lcp) of the two suffixes $T[\textit{SA}[i-1]..]$ and $T[\textit{SA}[i]..]$ (which are consecutive in lexicographic order). A variant of the $\textit{LCP}$ array is the *permuted* $\textit{LCP}$-*array*, $\textit{PLCP}$, defined as $\textit{PLCP}[i] = \textit{LCP}[\textit{ISA}[i]]$, i.e. the lcp values are stored in text order, rather than in $\textit{SA}$ order. We further define $\textit{LCPsum}(T)=\sum _{i=1}^{|T|} \textit{LCP}[i]$. $\textit{LCPsum}$ can be used as a measure of repetitiveness of strings, since the number of distinct substrings of *T* equals ${(|T|^2+|T|)}/{2} - \textit{LCPsum}(T)$. All these arrays can be computed in linear time in |*T*|, see e.g. [16, 20].

Given the suffix array $\textit{SA}$ of *T* and a substring *U* of *T*, the indices of all suffixes which have *U* as prefix appear consecutively in $\textit{SA}$. We refer to this interval as *U*-*interval*: the *U*-interval is $\textit{SA}[s..e]$, where $\{ \textit{SA}[s],\textit{SA}[s+1],\ldots ,\textit{SA}[e-1],\textit{SA}[e]\}$ are the starting positions of the occurrences of *U* in *T*.

Let ${{\mathcal {C}}} = \{S_1,\ldots ,S_m\}$ be a collection of strings (a set or multiset). The *generalized suffix array* $\textit{GSA}$ of ${{\mathcal {C}}}$ is defined as $\textit{GSA}[i]=(d,j)$ if $S_d[j..]$ is the *i*th suffix in lexicographic order among all suffixes of the strings from ${{\mathcal {C}}}$, where ties are broken by the document index *d*. The *GSA* can be computed in time ${{\mathcal {O}}}(N)$, where *N* is the total length of strings in ${{\mathcal {C}}}$ [1].

Let *R* and *S* be two strings. The *matching statistics of S with respect to R* is an array $\textit{MS}$ of length |*S*|, defined as follows. Let *U* be the longest prefix of suffix *S*[*i*..] which occurs in *R* as a substring, where the end-of-string character $\#$ of *R* is assumed to be different from, and smaller than that of *S*. Then $\textit{MS}[i] = (p_i,\ell _i)$, where $p_i=-1$ if $U=\varepsilon$, and $p_i$ is an occurrence of *U* in *R* otherwise, and $\ell _i = |U|$. (Note that $p_i$ is not unique in general.) We refer to *U* as the *matching factor*, and to the character *c* immediately following *U* in *S* as the *mismatch character*, of position *i*. For a collection ${{\mathcal {C}}} = \{S_1,\ldots ,S_m\}$ and a string *R*, the matching statistics of ${{\mathcal {C}}}$ w.r.t. *R* is simply the concatenation of $\textit{MS}_i$’s, where $\textit{MS}_i$ is the matching statistics of $S_i$ w.r.t. *R*. We will discuss matching statistics in more detail in “Compressed matching statistics” section.

For an integer array *A* of length *n* and an index *i*, the previous and next smaller values, $\textit{PSV}$ resp. $\textit{NSV}$, are defined as $\textit{PSV}(A, i) = \max \{i'< i: A[i'] < A[i]\}$ resp. $\textit{NSV}(A, i) = \min \{i' > i: A[i'] < A[i]\}$. Note that $\textit{PSV}$ resp. $\textit{NSV}$ is not defined for $i = 1$ resp. $i = n$. In *O*(*n*) preprocessing of *A*, a data structure of size $n \log _2(3 + 2\sqrt{2}) + o(n)$ bits can be built that supports answering arbitrary *PSV* and *NSV* queries in constant time per query [21].

Let *X* be a finite set of integers. Given an integer *x*, the predecessor of *x*, $\textit{pred}(x)$ is defined as the largest element smaller than *x*, i.e. $\textit{pred}_X(x) = \max \{y\in X \mid y \le x\}$. Using the y-fast trie data structure of Willard [22] allows answering predecessor queries in $O(\log \log |X|)$ time using *O*(|*X*|) space.

We are now ready to state our problem:

Problem statement: Given a string collection ${{\mathcal {C}}} = \{ S_1,\ldots , S_m\}$ and a reference string *R*, compute the generalized suffix array $\textit{GSA}$ of ${{\mathcal {C}}}$.

We will denote the length of *R* by *n* and the total length of strings in the collection by $N=\sum _{d=1}^m |S_d|$. As before, we assume that the end-of-string character $\#$ of *R* is strictly smaller than those of the strings in the collection ${{\mathcal {C}}}$. We are interested in those cases where $\textit{LCPsum}_R$ is small and the strings in ${{\mathcal {C}}}$ are very similar to *R*. If no reference string is given in input, we will take $S_1$ to be the reference string by default.

### Efficient suffix array construction

Currently, the best known and conceptually simplest linear-time suffix array construction algorithm is the SAIS algorithm by Nong et al. [16]. It cleverly combines, and further develops, several ideas used by previous suffix array construction algorithms, among these *induced sorting*, and use of a so-called *type array*, already used in [23, 24] (see also [5]).

Nong et al.’s approach can be summarized as follows: assign a type to each suffix, sort a specific subset of suffixes, and compute the complete suffix array by inducing the order of the remaining suffixes from the sorted subset. There are three types of suffixes, one of which constitutes the subset to be sorted first.

The definition of types is as follows (originally from [24], extended in [16]): Suffix *i* is *S*-*type* (smaller) if $T[i..] < T[i+1..]$, and *L*-*type* (larger) if $T[i..] > T[i+1..]$. An *S*-type suffix is $S^*$-*type* if *T*[*i*..] is *S*-type and $T[i-1..]$ is *L*-type. It is well known that assigning a type to each suffix can be done with a back-to-front scan of the text in linear time.

Now, if the relative order of the $S^*$-suffixes is known, then that of the remaining suffixes can be induced with two linear scans over the partially filled-in suffix array: the first scan to induce *L*-type suffixes, and the second to induce *S*-type suffixes. For details, see [16] or [1].

Another ingredient of SAIS, and of several other suffix array construction algorithms, is what we term the *metacharacter method*. Subdivide the string *T* into overlapping substrings, show that if two suffixes start with the same substring, then their relative order depends only on the remaining part; assign metacharacters to these substrings according to their rank (w.r.t. the lexicographic order, or some other order, depending on the algorithm), and define a new string on these metacharacters. Then the relative order of the suffixes of the new string and the corresponding suffixes starting with these specific substrings will coincide. In SAIS [16], so-called LMS-substrings are used, while a similar method is applied in prefix-free-parsing (PFP) [17]. Here we will apply this method using substrings starting in special positions which we term insert-heads, see “Comparing two suffixes via the enhanced CMS” and “Putting it all together” sections for details.

## Compressed matching statistics

Let *R*, *S* be two strings over $\Sigma$ and $\textit{MS}$ be the matching statistics of *S* w.r.t. *R*. Let $\textit{MS}[i] = (p_i,\ell _i)$. It is a well known fact that if $\ell _i>0$, then $\ell _{i+1} \ge \ell _i-1$. This can be seen as follows. Let *U* be the matching factor of position *i*, and $p_i$ an occurrence of *U* in *R*. Then $U' = U[2..\ell _i]$ is a prefix of $S[i+1..]$ of length $\ell _i-1$, which occurs in position $p_i+1$ of *R*.

Let us call a position *j* a *head* if $\ell _j > \ell _{j-1}-1$, and a sequence of the form $(x,x-1,x-2,\ldots )$, of length at most $x-1$, a *decrement run*, i.e. each element is one less than the previous one. Using this terminology, we thus have that the sequence $L=(\ell _1,\ell _2,\ldots , \ell _n)$ is a concatenation of decrement runs, i.e. *L* has the form $(x_1,x_1-1,x_1-2,\ldots , x_2,x_2-1,x_2-2,\ldots ,\ldots ,x_k,x_k-1,x_k-2,\ldots )$, with each $x_j=\ell _j$ for some head *j*. We can therefore store the matching statistics in compressed form as follows:

### **Definition 1**

(*Compressed matching statistics*) Let *R*, *S* be two strings over $\Sigma$, and $\textit{MS}$ be the matching statistics of *S* w.r.t. *R*. The *compressed matching statistics* (*CMS*) *of* *S* *w.r.t.* *R* is a data structure storing $(j,\textit{MS}[j])$ for each head *j*, and a predecessor data structure on the set of heads *H*.

We can use $\textit{CMS}$ to recover all values of $\textit{MS}$:

### **Lemma 1**

*Let *$1\le i\le |S|$*. Then *$\textit{MS}[i] = (p_j+k,\ell _j-k)$*, where* $j = \textit{pred}_H(i)$ *and* $k = i-j$.

### ***Proof***

Let $\ell _i$ be the length of the matching factor of *i*. Since there is a matching factor of length $\ell _j$ starting in position *j* in *S*, this implies that $\ell _i \ge \max (0,\ell _j-k)$. If $\ell _i$ was strictly greater than $\ell _j-k$, this would imply the presence of another head between *j* and *i*, in contradiction to $j = \textit{pred}_H(i)$. Since an occurrence of the matching factor $U_j$ of *j* starts in position $p_j$ of *R*, therefore the matching factor $U'=U[k+1..\ell _j]$ of *i* has an occurrence at position $p_j+k$. $\square$

### *Example 1*

Consider the reference $R=\texttt{TGATGGCACAGATACT\#}$ and $S=$ $\texttt{GATGGCACATTGATGG\$}$. The *CMS* of *S* w.r.t. *R* is: (1, 2, 9), (9, 12, 2), (11, 1, 6), see Table 1.

**Table 1:** Example for the matching statistics and the data for the *CMS* and the *eCMS*

*i*	1	2	3	4	5	6	7	8	9	10	11	12	13	14	15	16	17
*R*	T	G	A	T	G	G	C	A	C	A	G	A	T	A	C	T	#
*S*	G	A	T	G	G	C	A	C	A	T	T	G	A	T	G	G	$
$$p_i$$	2	3	4	5	6	7	8	9	12	13	1	2	3	4	5	6	-1
$$\ell _i$$	9	8	7	6	5	4	3	2	2	1	6	5	4	3	2	1	0
Head	$$\checkmark$$								$$\checkmark$$		$$\checkmark$$						
$$q_i$$	2	3	4	5	6	7	8	9	3	4	1	2	3	4	5	11	17
i-head	$$\checkmark$$								$$\checkmark$$		$$\checkmark$$					$$\checkmark$$	$$\checkmark$$

In the first two rows, we give $\textit{MS}$ of *S* w.r.t. *R*, where $\textit{MS}[i]=(p_i,\ell _i)$. In row 3, we mark the heads (for the *CMS*). In rows 4, we give the position $q_i$, defined by *ip*(*i*), i.e. $q_i=\textit{SA}_R[ip(i)]$, where *ip*(*i*) is the insert-point of suffix *S*[*i*..] in the suffix array of *R*. In row 5, we mark the insert-heads (for the *eCMS*)

From Lemma 1 and the properties of the predecessor data structure on the set of heads we get:

### **Proposition 1**

*Let* *R*, *S* *be two strings over* $\Sigma$.* We can store the matching statistics of S w.r.t.* *R* *in* ${{\mathcal {O}}}(\chi )$ *space such that any entry* $\textit{MS}[i]$*, for *$1\le i \le |S|$*, can be accessed in* ${{\mathcal {O}}}(\log \log \chi )$ *time, where *$\chi =|H|$ *is the number of heads.*

For some statistics on the number $\chi$ of heads, see the end of “Enhancing the CMS” section.

### Enhancing the CMS

Let *R*, *S* be two strings over $\Sigma$, and $\textit{MS}$ the matching statistics of *S* w.r.t. *R*. We now assume that all characters that occur in *S* also occur in *R* (see “Implementation details” section). Let $\textit{SA}_R$ be the suffix array of *R*. For position *i* of *S*, let $U\ne \varepsilon$ be the matching factor and *c* the mismatch character of *i*. We want to compute the position that the suffix *S*[*i*..] would have in $\textit{SA}_R$ if it was present. To this end, we define the *insert point* of *i*, *ip*(*i*), as follows:

$$\begin{aligned} ip(i) = {\left\{ \begin{array}{ll} 1 &{} \hbox { if}\ U=\varepsilon ,\\ \max \{ j \mid U \text { is a prefix of } R[\textit{SA}_R[j]..] \quad \\ \qquad \text { and } R[\textit{SA}_R[j]..] < Uc\} &{} \text { if this set is non-empty,}\\ \min \{j \mid U \text { is a prefix of } R[\textit{SA}_R[j]..]\} &{} \text { otherwise.} \end{array}\right. } \end{aligned}$$

In other words, the insert point is the lexicographic rank, among all suffixes of *R*, of the next smaller occurrence of *U* in *R* if such an occurrence exists, and of the smallest occurrence of *U* in *R* otherwise. Note that case 1 (where $U=\varepsilon$) only happens for end-of-string characters. The insert point is well-defined for every *i* because $\#$ is smaller than all other characters, including other end-of-string characters. Observe that the insert point of *i* always lies within the *U*-interval of $\textit{SA}_R$. For an example, see Table 2.

**Table 2:** Details of computation of the matching statistics from Table 1.

	*i*	$\textit{SA}_R$	$R[{\textit{SA}_R[i]}..]$
	1	17	#
	2	8	ACAGATACT#
	3	14	ACT#
	4	10	AGATACT#
	5	12	ATACT#
$\rightarrow$	6	3	ATGGCACAGATACT#
	7	7	CACAGATACT#
	8	9	CAGATACT#
	9	15	CT#
	10	11	GATACT#
	11	2	GATGGCACAGATACT#
	12	6	GCACAGATACT#
	13	5	GGCACAGATACT#
	14	16	T#
	15	13	TACT#
$\rightarrow$	16	1	TGATGGCACAGATACT#
	17	4	TGGCACAGATACT#

We underline the matching factors for the indices $i=9$ (matching factor $\texttt{AT}$, mismatch character $\texttt{T}$) and 11 (matching factor $\texttt{TGATGG}$, mismatch character $\mathtt{\$}$). The arrows represent the insert-points

We will later use the insert points to bucket suffixes. First we need to slightly change the definition of our compressed matching statistics. We will add more information to the heads: we add the mismatch character and replace the position entry $p_i$, which gives just some occurrence of the matching factor, by the specific occurrence $q_i$ given by the insert point. This will imply adding more heads, so our data structure may increase in size.

To this end, we define *j* to be an *insert-head* if $\textit{SA}_R[ip(j)] \ne \textit{SA}_R[ip(j-1)]+1$. Note that, in particular, all heads are also insert-heads, but it is possible to have insert-heads *j* which are not heads, namely where $\ell _j = \ell _{j-1}-1$.

#### **Definition 2**

(*Enhanced compressed matching statistics*) Let *R*, *S* be two strings over $\Sigma$. Define the *enhanced matching statistics* as follows: for each $1\le i \le |S|$, let $\textit{ems}(i) = (q_i,\ell _i,x_i,c_i)$, where $q_i = \textit{SA}_R[ip(i)]$, $\ell _i$ is the length of the matching factor *U* of *i*, $c_i$ is the mismatch character, and $x_i\in \{S,L\}$ indicates whether $Uc_i$ is smaller (S) or greater (L) than $R[q_i..]$. The *enhanced compressed matching statistics* (*eCMS*) *of* *S* *w.r.t.* *R* is a data structure storing $(j,\textit{ems}(j))$ for each insert-head *j*, and a predecessor data structure on the set of insert-heads $H'$.

#### *Example 2*

Continuing with Example 1, the enhanced *CMS* of *S* w.r.t. *R* is: $(1,2,9,L,\texttt{T})$, $(9,3,2,L,\texttt{T})$, $(11,1,6,S,\mathtt{\$}),$ $(16,11,1,S,\mathtt{\$})$, $(17,17,0,L,\mathtt{\$})$, see Table 1.

We will need some properties of the insert point in the following:

#### Observation 1

Let *ip*(*i*) be the insert point of *i*, and $\textit{ems}(i) = (q_i,\ell _i,x_i,c_i)$.

1. $ip(i) = ip(i')$ if and only if $q_i = q_{i'}$,
2. if $$x_i=S$$ then $$R[\textit{SA}_R[ip(i)-1]..]< S[i..] < R[\textit{SA}_R[ip(i)]..] = R[q_i..]$$,
3. if $$x_i=L$$ then $$R[q_i..] = R[\textit{SA}_R[ip(i)]..]< S[i..] < R[\textit{SA}_R[ip(i)+1]..]$$.

The enhanced CMS can be used in a similar way as the CMS to recover the enhanced matching statistics (including the matching statistics) of each *i*. Denote by $\textit{i-head}(i)$ the next insert-head to the left of *i*, i.e. $\textit{i-head}(i) = \max \{j\le i \mid j \text { is an insert-head}\}$. Note that $\textit{i-head}(i) = \textit{pred}_{H'}(i)$.

#### **Lemma 2**

*Let* $1\le i \le |S|$*, let *$\textit{eCMS}$ *be the enhanced CMS of S* *w.r.t.* *R*. *Let* $j = \textit{i-head}(i)$, $k=i-j$, *and *$\textit{ems}(j) = (q_j,\ell _j,x_j,c_j)$. *Then* $\textit{ems}(i) = (q_j+k,\ell _j-k,x_j,c_j)$,* and* $ip(i) = \textit{ISA}_R[q_j+k]$.* In particular, *$q_j+k$ *is an occurrence and* $\ell _j-k$ *is the length of the matching factor of* *i* *(in other words, the matching statistics entry* $\textit{MS}[i]$).

#### ***Proof***

Analogous to Lemma 1, resp. straightforward from the definitions. $\square$

Similarly to the *CMS* (cp. Prop 1), the enhanced *CMS* allows access to all values for every index *i*, using space ${{\mathcal {O}}}(\chi ')$ and time ${{\mathcal {O}}}(\log \log \chi ')$, where $\chi '=|H'|$ is the number of insert-heads. Again, this is due to the fact that the predecessor data structure on the set $H'$ of insert-heads allows retrieving $\textit{pred}_{H'}(i) =\textit{i-head}(i)$ in ${{\mathcal {O}}}(\log \log |H'|)$ time, and the values of $\textit{ems}(i)$ can then be computed in ${{\mathcal {O}}}(1)$ time (Lemma 2).

We close this subsection by remarking that for a collection of similar genomes, one can expect the number of heads to be small. Indeed, on a 500MB viral genome data set (see “Datasets” section) containing approximately 10,000 SARS-cov2 genomes, we observed the number of heads to be 5,326,226 (100x less than the input size) and the number of insert heads to be 6,537,294.

### Computing the CMS

It is well known that the matching statistics of *S* w.r.t. *R* can be computed in time $O(|R| + |S|\log \sigma )$ and *O*(|*R*|) space by using, for example, the suffix tree of *R*, as described in Chang and Lawler’s original paper [18]. Since then, several authors have described similar algorithms for computing matching statistics, all focussed on reducing space requirements via the use of compressed indexes instead of the suffix tree [3, 25, 26]. These algorithms all incur the slowdowns typical of compressed data structures.

In our setting, where end-to-end runtime is the priority, it is the speed at which the matching statistics can be computed (rather than working space) that is paramount. Moreover, because the size of the reference is generally small relative to the total length of all the strings $S_i \in {{\mathcal {C}}}$, we have some freedom to use large index data structures on *R* to compute the matching statistics, without overall memory usage getting out of hand. With these factors in mind, we take the following approach to computing CMS. The algorithm is similar to that of Chang and Lawler, but makes use of array-based data structures rather than the suffix tree.

Recall that, given the suffix array $\textit{SA}_R$ of string *R* and a substring $\textit{Y}$ of *R*, the $\textit{Y}$-interval is the interval $\textit{SA}_R[s..e]$ that contains all suffixes having $\textit{Y}$ as a prefix.

#### **Definition 3**

(*Right extension and left contraction*) For a character *c* and a string $\textit{Y}$, the computation of the $\textit{Yc}$-interval from the $\textit{Y}$-interval is called a *right extension* and the computation of the $\textit{Y}$-interval from $\textit{cY}$-interval is called a *left contraction*.

We remark that a left contraction is equivalent to following a (possibly implicit) suffix link in the suffix tree of *R* and a right extension is a downward movement (either to a child or along an edge) in the suffix tree of *R*.

Given a *Y*-interval, because of the lexicographical ordering on the $\textit{SA}_R$, we can implement a right extension to a $\textit{Yc}$-interval in $O(\log |R|)$ time by using a pair of binary searches (with *c* as the search key), one to find the lefthand end of the $\textit{Yc}$-interval and another to find the righthand end. If a right extension is empty then there are no occurrences of $\textit{Yc}$ in *R*, but we can have the binary search return to us the insert point where it would have been in $\textit{SA}_R$.

On the other hand, given a *cY*-interval, $\textit{SA}_R[s..e]$, we can compute the *Y*-interval (i.e. perform a left contraction) in the following way. Let the target *Y*-interval be $\textit{SA}_R[x..y]$. Observe that both $\textit{SA}_R[s]+1$ and $\textit{SA}_R[e]+1$ must be inside the *Y*-interval, $SA_R[x..y]$—that is, $s' = \textit{ISA}_R[\textit{SA}_R[s]+1] \in [x..y]$ and $e' = \textit{ISA}_R[\textit{SA}_R[e]+1] \in [x..y]$. To finish computing $\textit{SA}_R[x..y]$, note that $[s'..e']$ is contained in $[x..y]$, but there may be occurences of *Y* which come before $s'$ or after $e'$ in $\textit{SA}_R$. For this, we use a variant of $\textit{PSV}/\textit{NSV}$-queries: $\textit{PSV}(A,i,\ell)= \max\{ i'\leq i : A[i'] < \ell\}$ and $\textit{NSV}(A,i,\ell) = \min \{ i'\geq i : A[i'] < \ell\}$. Then $\textit{SA}_R[x..y] =\textit{SA}_R[\textit{PSV}(\textit{LCP}_R,s', |Y|)..\textit{NSV}(\textit{LCP}_R,e'+ 1,|Y|)-1]$.

With these ideas in place, we are ready to describe the matching statistics algorithm. We first compute $\textit{SA}_R$, $\textit{ISA}_R$, and $\textit{LCP}_R$ for *R* and preprocess $\textit{LCP}_R$ for $\textit{NSV}/\textit{PSV}$ queries. The elements of the $\textit{MS}$ will be computed in left-to-right order, $\textit{MS}[1], \textit{MS}[2], \ldots , \textit{MS}[|S|]$. Note that this makes it trivial to save only the heads (or iheads) and so compute the CMS (or eCMS) instead. To find $\textit{MS}[1]$ use successive right extensions starting with the interval $SA_R[1..|R|]$, searching with successive characters of *S*[1..] until the right extension is empty, at which point we know $\ell _1$ and $p_1$. At a generic step in the algorithm, immediately after computing $\textit{MS}[i]$, we know the interval $\textit{SA}_R[s_i..e_i]$ containing all the occurrences of $R[p_i..p_i+\ell _i-1]$. To compute $\textit{MS}[i+1]$ we first compute the left contraction of $\textit{SA}_R[s_i..e_i]$, followed by as many right contractions as possible until $\ell _{i+1}$ and $p_{i+1}$ are known.

When profiling an implementation of the above algorithm, we noticed that very often the sequence of right extensions ended with a singleton interval (i.e., an interval of size one) and so was the interval reached by the left contraction that followed. In terms of the suffix tree, this corresponds to the match between *R* and the current suffix of $S_i$ being inside a leaf branch. This frequently happens on genome collections because each sequence is likely to have much longer matches with other sequences (in this case with *R*) than it does with itself (a single genome tends to look fairly random, at least by string complexity measures).

A simple heuristic to exploit this phenomenon is to compare $\ell _i$ to the maximum value in the entire $\textit{LCP}_R$ array of *R* immediately after $\textit{MS}[i]$ has been computed. If $\ell _i-1 > \max (\textit{LCP}_R)$ then $\textit{ISA}_R[p_i+1]$ will also be inside a leaf branch (i.e., the left contraction will also be a singleton interval), and so the left contraction can be computed trivially as $\textit{ISA}_R[p_i+1]$—with no subsequent $\textit{NSV}/\textit{PSV}$ queries or access to $\textit{LCP}_R$ required to expand the interval. Although this gives no asymptotic improvement, there is potential gain from the probable cache miss(es) avoided by not making random accesses to those large data structures.

On a viral genome data set (see “Experiments” section), $\max (\textit{LCP}_R)$ was 14, compared to an average $\ell _i$ value of over 1, 100, and this heuristic saved lots of computation. On a human chromosome data set, however, $\max (\textit{LCP}_R)$ was in the hundreds of thousands, and so we generalized the trick in the following way. We divide the LCP array up into blocks of size *b* and compute the maximum of each block. These maxima are stored in an array *M* of size |*R*|/*b*, and *b* is chosen so that *M* is small enough to comfortably fit in cache. Now, when transitioning from $\textit{MS}[i]$ to $\textit{MS}[i+1]$, if $\ell _i > M[\textit{ISA}_R[p_i+1]/b]$ then there is a single match corresponding to $\textit{MS}[i+1]$, which we compute with right extensions. This generalized form of the heuristic has a consistent and noticeable effect in practice. For a 500MB viral genome data set its use reduced CMS computation from 12.23 s to 2.34 s. On the human chromosome data set the effect is even more dramatic: from 76.50 s down to 7.14 s.

## Comparing two suffixes via the enhanced CMS

We will now show how to use the enhanced CMS of the collection ${{\mathcal {C}}}$ w.r.t. *R* to define a partial order on the set of suffixes of strings in ${{\mathcal {C}}}$ (Prop. 2), and how to break ties when the entries are identical (Lemma 5). These results can then be used either directly to determine the relative order of any two of the suffixes (Prop. 3), or as a way of inducing the complete order once that of the subset of the insert-heads has been determined (Prop. 4).

We will prove Prop. 2 via two lemmas. Recall that in the *eCMS* we only have the entries referring to the insert-heads; however, Lemma 2 tells us how to compute them for any position.

### **Lemma 3**

*Let* $1\le d,d' \le m$ *and* $1\le i\le |S_d|$, $1\le i'\le |S_{d'}|$.* If *$ip(d,i) < ip(d',i')$, *then* $S_d[i..] < S_{d'}[i'..]$.

### ***Proof***

If $ip(d',i')-ip(d,i)>1$, then there exists an index *j* s.t. $ip(d,i)<j<ip(d',i')$, and therefore $S_d[i..]< R[\textit{SA}_R[ip(d,i)+1]..] \le R[\textit{SA}_R[j]..] \le R[\textit{SA}_R[ip(d',i')-1]..] < S_{d'}[i'..]$. Now let $ip(d',i') = ip(d,i)+1$. If $x_{d,i}=S$, then $S_d[i..]<R[\textit{SA}_R[ip(d,i)]..] = R[\textit{SA}_R[ip(d',i')-1]..] < S_{d'}[i'..]$, by Obs. 1. Similarly, if $x_{d',i'}=L$, then $S_d[i..]<R[\textit{SA}_R[ip(d,i)+1]..] = R[\textit{SA}_R[ip(d',i')]..] < S_{d'}[i'..]$. Finally, let $x_{d,i}=L$ and $x_{d',i'}=S$. Then $R[\textit{SA}_R[ip(d,i)]..]< S_d[i..],S_{d'}[i'..] < R[\textit{SA}_R[ip(d,i)+1]..] = R[\textit{SA}_R[ip(d',i')]..]$. Let *U* be the matching factor of (*d*, *i*), $U'$ that of $(d',i')$, and $V = \textit{lcp}(U,U')$, the longest common prefix of the two. *V* cannot be equal to $U'$ because then $U'$ would be a proper prefix of *U*, but $ip(d',i')$ is the smallest occurrence in *R* of $U'$. If $V=U$, then *U* is a proper prefix of $U'$, and by definition of $ip(d',i')$, the character following *U* in $U'$ is strictly greater than the mismatch character $c_i$ of (*d*, *i*). Finally, if *V* is a proper prefix both of *U* and of $U'$, then the character following *V* in *U* is smaller than the one following *V* in $U'$, therefore $U<U'$. Since *U* is a prefix of $S_d[i..]$ and $U'$ is a prefix of $S_{d'}[i'..]$, and neither is prefix of the other, this implies $S_d[i..] < S_{d'}[i'..]$. $\square$

### **Lemma 4**

*Let *$1\le d,d' \le m$ *and* $1\le i\le |S_d|$, $1\le i'\le |S_{d'}|$, *and* $ip(d,i) = ip(d',i')$.

1. If $\ell _{d,i} < \ell _{d',i'}$ and $x_{d,i}=S$, then $S_d[i..] < S_{d'}[i'..]$.
2. If $\ell _{d,i} < \ell _{d',i'}$ and $x_{d,i}=L$, then $S_{d'}[i'..] < S_d[i..]$.
3. If $\ell _{d,i} = \ell _{d',i'}$ and $x_{d,i}=S$ and $x_{d',i'}=L$, then $S_d[i..] < S_{d'}[i'..]$.
4. If $\ell _{d,i} = \ell _{d',i'}$ and $x_{d,i}=x_{d',i'}$ and $c_{d,i}<c_{d',i'}$, then $S_d[i..] < S_{d'}[i'..]$.

### ***Proof***

*1.,2.:* Let *U* be the matching factor of *i*, and $U'$ that of $i'$. Since $\ell _{d,i}<\ell _{d',i'}$, this implies that *U* is a proper prefix of $U'$. If $x_{d,i}=S$, then the mismatch character $c_{d,i}$ is smaller than the character following *U* in $U'$, therefore $S_d[i..] < S_{d'}[i'..]$. If $x_{d,i}=L$, then it is greater, and thus $S_{d'}[i'..] < S_d[i..]$. *3.* follows directly from Observation 1, since now $S[i..]< R[\textit{SA}_R[ip(i)]..] < S[i'..]$. *4.: * Now both suffixes start with the same matching factor *U*, followed by different mismatch characters, which define their relative order. $\square$

These two lemmas in fact imply the following:

### **Proposition 2**

*The relation defined in Lemmas* 3*and* 4*is a partial order of the suffixes of strings in* ${{\mathcal {C}}}$,* of which the lexicographic order is a refinement.*

### ***Proof***

It follows from Lemmas 3 and 4 that the lexicographic order is a refinement of the relation defined. This, on the other hand, implies that it is a partial order. $\square$

What happens if two suffixes $S_d[i..]$ and $S_{d'}[i'..]$ have the same values of the enhanced matching statistics, i.e. $\textit{ems}(d,i) = \textit{ems}(d',i')$? The next lemma says that in this case, the relative order of the two suffixes is decided by the relative order of the heads preceding their respective mismatch characters.

### **Lemma 5**

*Let* $1\le d,d' \le m$ *and* $1\le i\le |S_d|$, $1\le i'\le |S_{d'}|$. *If* $ip(d,i) = ip(d',i')$, $\ell _{d,i} = \ell _{d',i'}$, $x_{d,i}=x_{d',i'}$, *and* $c_{d,i}=c_{d',i'}$, *then* $S_d[i..] < S_{d'}[i'..]$ *if and only if* $S_d[j..] < S_{d'}[j'..]$, *where* $(d,j) = \textit{i-head}(d,i+\ell _i)$ *and* $(d',j') = \textit{i-head}(d',i'+\ell _{i'})$.

### ***Proof***

We will prove that the relative position of the insert-head of *i*’s and $i'$’s mismatch character is the same, i.e. that $j-i = j'-i'$. The claim then follows.

First note that $j>i$. This is because the matching factor of position *i* ends in position $i+\ell _{d,i}-1$, so there must be a new insert-head after *i* and at most at $i+\ell _{d,i}$, the position of the mismatch character. Similarly, $j'>i'$. The fact that $j=\textit{i-head}(i+\ell _{d,i})$ implies that there is a matching factor starting in position *j* which spans the mismatch character $c=c_{d,i}=c_{d',i'}$. Let’s write *Vc* for the prefix of length $i+\ell _{d,i}-j$ of this matching factor. *V* is a suffix of the matching factor *U* of position *i*, but *Vc* is not. However, *Vc* is also a prefix of $S_{d'}[i'..]$. Therefore, $j'=i'+(j-i)$ is also an insert-head in $S_{d'}$. An analogous argument shows that any insert-head between $i'$ and $i'+\ell _{d',i'}$ in $S_{d'}$ is also an insert-head in $S_d$, in the same relative position. $\square$

### **Proposition 3**

*Let *$R,S_1,\ldots ,S_m$ *be strings over* $\Sigma$*. Using the enhanced* *CMS* of ${{\mathcal {C}}}=\{S_1,\ldots ,S_m\}$ *w.r.t. R*, *we can decide, for any* $1\le d,d' \le m$ *and* $1\le i\le |S_d|$, $1\le i'\le |S_{d'}|$,* the relative order of* $S_d[i..]$ *and* $S_{d'}[i'..]$ *in* ${{\mathcal {O}}}(\log \log \chi '\cdot \max _d \{\text {no.\ of insert-heads of } S_d\})$ *time*.

### ***Proof***

Let $(d,j) = \textit{i-head}(d,i+\ell _i)$ and $(d',j') = \textit{i-head}(d',i'+\ell _{i'})$. From Lemma 2 we get the four *eCMS*-entries of (*d*, *i*) and $(d',i')$, namely the insert positions $q_i$ resp. $q_{i'}$, the length of the matching factor, whether the mismatch characters is smaller or larger, and the mismatch character itself. If any of these differ for the two suffixes, then Lemmas 3 and 4 tell us their relative order. This check takes ${{\mathcal {O}}}(1)$ time. Otherwise, Lemma 5 shows that the relative order is determined by the next relevant heads. Iteratively applying the three lemmas, in the worst case, takes us through all heads for the strings $S_d$ and $S_{d'}$. $\square$

Instead of using Prop. 3, we will use these lemmas in the following way. We will first sort only the insert-heads. The following proposition states that this suffices to determine the order of any two suffixes in constant time.

### **Proposition 4**

*Given the insert-heads in sorted order, the relative order of any two suffixes can be determined in* ${{\mathcal {O}}}(\log \log \chi ')$ *time, where* $\chi '$ *is the number of insert-heads*.

### ***Proof***

Follows from Lemmas 3, 4, and 5, since all checks take constant time, and each of the two predecessor queries take ${{\mathcal {O}}}(\log \log \chi ')$ time. $\square$

## Putting it all together

A high-level view of our algorithm is as follows. We first partially sort the insert-heads, then use this partial sort to generate a new string, whose suffixes we sort with an existing suffix sorting algorithm. This gives us a full sort of the insert heads. We then use this to sort the $S^*$-suffixes of the collection. Finally, we induce the remaining suffixes of the collection using the $S^*$-suffixes. We next give a schematic description of the algorithm.

**Algorithm 1:**
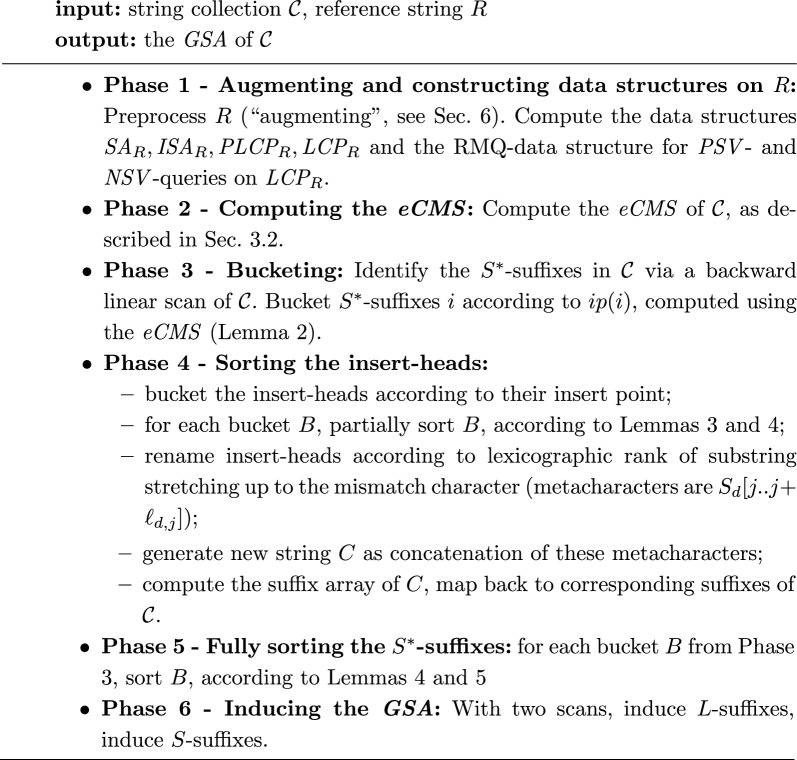
.

We next give a worst-case asymptotic analysis of the algorithm.

### **Proposition 5**

*Algorithm 1 computes the* *GSA* *of a string collection* ${{\mathcal {C}}}$ *of total length* *N in worst-case time* ${{\mathcal {O}}}(N\log N)$.

### ***Proof***

Let $|R|=n$. Phase 1 takes ${{\mathcal {O}}}(n+N)$ time, since constructing all data structures on *R* can be done in linear time in *n* and scanning the collection ${{\mathcal {C}}}$ takes time ${{\mathcal {O}}}(N)$. Phase 2 takes time ${{\mathcal {O}}}(N\log n)$ using the algorithm from “Computing the CMS” section. In Phase 3, identifying the $S^*$ suffixes, takes time ${{\mathcal {O}}}(N)$. Since at this point, the $\textit{eCMS}$ is in text-order, identifying $\textit{i-head}(i)$ takes constant time, also computing the insert-point takes constant time, so altogether ${{\mathcal {O}}}(N)$ time. In Phase 4, all steps are linear in $\chi '$, the number of insert-heads, including the partial sort of the buckets, since this can be done with radix-sort (three passes over each bucket), so this phase takes time ${{\mathcal {O}}}(\chi ')$. Phase 5 takes time ${{\mathcal {O}}}(|B|\log |B|)$ for each bucket *B*, thus ${{\mathcal {O}}}(N\log |B_{\max }|)$ for the entire collection, where $B_{\max }$ is a largest bucket. Since all strings in the collection are assumed to be highly similar to the reference, the size of the buckets can be expected to vary around the number of strings in the collection *m*; however, in the worst case the largest bucket can be $\Theta (N)$. Finally, Phase 6 takes linear time ${{\mathcal {O}}}(N)$. Altogether, the running time is dominated by Phase 5, ${{\mathcal {O}}}(N\log N)$. $\square$

## Implementation details

In Phase 1, the augmentation step involves, for every character *c* not occurring in *R* but occurring in ${{\mathcal {C}}}$, appending $c^{n_c}$ to *R*, where $n_c$ is the length of the longest run of *c* in ${{\mathcal {C}}}$. This avoids having 0-length entries in the matching statistics and is necessary in order to have a well defined insert point *ip*.

To compute $\textit{SA}_R$ in Phase 1, we use libsais [27] as implemented by Ilya Grebnov, a well-engineered version of SAIS [16]. We chose this implementation due to its consistent speed on many different inputs. For the computation of $\textit{PLCP}_R$ and $\textit{LCP}_R$, the same tool offers functions based on the $\Phi$ method [20]. We constructed the data structure of Cánovas and Navarro [28] for NSV/PSV queries on the LCP array, as it has low space overheads and was fast to query and initialize.

For the predecessor data structure, we use the following two-layered approach in practice (rather than [22]). We sample every *b*th head starting position and store these in an array. In a separate array we store a differential encoding of all head positions. The array of differentially encoded starting positions takes 32 bits per entry. Predecessor search for a position *x* proceeds by first binary searching in the sampled array to find the predecessor sample at index *i* of that array. We then access the differentially encoded array starting at index *ib* and scan, summing values until the cumulative sum is greater than *x*, at which point we know the predecessor. This takes $O(\log (\chi '/b) + b)$ time, where $\chi '$ is the number of insert-heads.

For Phase 4, when we have to sort *C* (the concatenation of metacharacters representing partially sorted heads), we use another function from libsais that handles integer alphabets.

### Parallel implementation

In Phase 1, for building the data structures of *R*, we use again functions from libsais, but this time with multithreading enabled.

We parallelized Phase 2, which consists in computing the enhanced matching statistics. We start by storing after the first scan each sequence boundaries. With this additional information, we can distribute evenly the sequences to the threads. Because we know that at the end of each sequence we have a separator, there is no extra boundary checking for the computation of the matching statistics.

In Phase 3, where we pre-bucket S*-suffixes based on their insert point, we allocate a thread-local buffer to count the frequencies for each bucket. Then, we perform a global prefix sum to get the correct positions for each thread. Ultimately, the writes can be made concurrently without having to lock the bucket counter.

In Phase 4, both the partial sort and computing *C* and its $\textit{SA}$ are easily parallelized by assigning different buckets to multiple threads.

Similarly, when we sort S*-suffixes in Phase 5 we assign each bucket to one of the different threads in parallel.

Finally, Phase 6—inducing the final suffix array—is the most difficult part of the algorithm to parallelize. As already detailed in the literature [29–31], in this phase only a partial parallelization can be achieved, due to the intrinsic sequential nature of induced sorting. More specifically, we use some helper threads to fetch in batch the characters preceding the suffixes in a specific range. Then, having stored this information in a buffer, we induce sequentially the correct position of suffixes.

## Experiments

We implemented our algorithm for computing the generalized suffix array in C++. Our prototype implementation, sacamats, is available at https://github.com/fmasillo/sacamats. The experiments were conducted on a desktop equipped with 64GB of RAM DDR4-3200MHz and an Intel(R) Core(R) i9-11900 @ 2.50GHz (with turbo speed @ 5GHz) with 16MB of cache. The operating system was Ubuntu 22.04 LTS, the compiler used was g++ version 11.3.0 with options -std=c++20 -O3 -funroll-loops -march=native enabled.

### Tools compared

In the experiments reported on below, we compared $\texttt{sacamats}$ to the following seven well-known suffix array construction tools, which represent the state of the art.

1. divsufsort [14], a tool implemented by Mori [13] that was considered, until recently, one of the fastest general-purpose SACAs. It is perhaps the most widely used tool in bioinformatics-related libraries.
2. sais-lite [15], also implemented by Yuta Mori, this tool implements the well-known SAIS algorithm by Nong et al. [16].
3. gsacak [32], an extension of the SACA-K algorithm [33] to a collection of strings.
4. big-bwt [17], a tool computing the $\textit{BWT}$ and the suffix array, designed specifically for highly repetitive data. We used the default parameters (-w = 10, -p = 100) and the -f flag to parse fasta files as input. The standard implementation streams to disk the *BWT* and the $\textit{SA}$. We made slight changes to the big-bwt code to enable storing the $\textit{SA}$ in main memory and also to skip the *BWT* being written to disk (both for serial and parallel implementations) for a fair comparison with the other tools.
5. gsaca-ds [34], an implementation of the GSACA algorithm by Baier [35]. This is the first non-recursive linear algorithm for suffix array construction. It is divided into two phases, first grouping suffixes into specific groups, and then using this information to complete sorting. This implementation uses integer sorting for both phases (hence the name double-sort). This tool offers four serial variants and three parallel variants. It was chosen as a competitor due to its good performance on repetitive data.
6. lfgsaca [36], another implementation of the GSACA algorithm. Again, it has been proven to be very fast on repetitive data.
7. libsais [27], the current fastest tool based on SAIS, implemented by Ilya Grebnov. It has not yet appeared in a peer-reviewed paper, but is available for download.

### Datasets

For our tests, we used two publicly available datasets, one consisting of copies of human chromosome 19 from the 1000 Genomes Project [37], and the other consisting of copies of SARS-CoV2 genomes taken from NCBI Datasets.^1^ The first dataset contains only characters A, C, G, T and N (thus, $\sigma =5$), while the second dataset contains also IUPAC codes ($\sigma =14$). For further details, see Table 3.

**Table 3:** Datasets used in the experiments

Name	Description	$\sigma$	No. of sequences	Ref. sequence length	*r*
chr19	Human Chromosome 19	5	103	59,126,939	33,799,549
sars-cov2	SARS-CoV2 genome	14	205,813	29,783	6,207,939

In column 3, we specify the alphabet size $\sigma$, in column 4 the number of sequences in the dataset, in column 5 the reference sequence length, and in column 6 the number of runs *r* in the *BWT*. The total dataset has size 6 GB

For both datasets, we selected subsets of different sizes in order to study the scalability of our algorithm, and for comparison with other tools. The sizes are 250 MB, 500 MB, 750 MB, 1 GB, 2 GB, 4 GB, and 6 GB.

We further computed several parameters which impact on the efficiency of the different algorithms, on the full datasets (size 6 GB), as well as on a subset of size 500 MB: the number *r* of runs of the BWT, the number of $S^*$-suffixes, and the number of i-heads. For details, see Table 4. We observe that, on all datasets, the number of i-heads is around 100 times less than the input size.

**Table 4:** Different parameters computed on a 500 MB subset of data, respectively on the whole dataset

Dataset	*r*	No. of $S^*$-suffixes	No. of i-heads
chr19 500 MB	32,018,267	129,130,084	4,220,033
sars-cov2 500 MB	377,437	143,672,321	6,537,294
chr19 6 GB	33,799,549	1,553,011,435	50,088,865
sars-cov2 6 GB	6,207,939	1,696,153,792	89,449,086

Even though the two real-life datasets have different characteristics (e.g., the average length of the sequences is around 59 million vs. 30 thousand), the parameters that influence our algorithm’s performance, namely, the number of $S^*$-suffixes and the number of i-heads, are similar. This is different from the number *r* of the BWT-runs, which, in collections of highly similar sequences, tends to be lower on collections of many short strings, such as sars-cov2.

For our final experiment, we used simulated data to study the effect on our algorithm of decreasing similarity within the sequence collection (see “Effect of repetitiveness on running time” section).

### Results

**Fig. 1:**
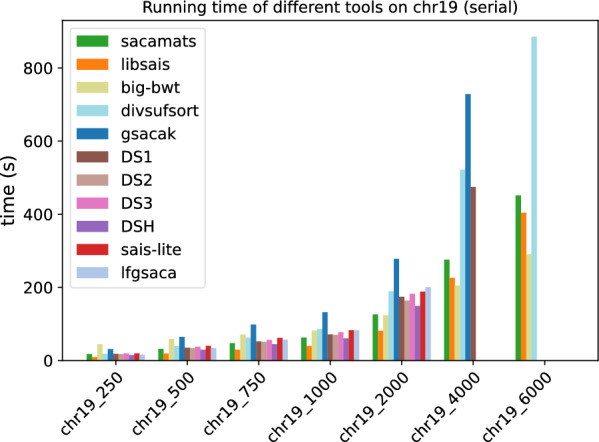
Comparison of running times of different tools (see “Tools compared” section) on subsets of varying length of the Chromosome 19 dataset (serial implementations)

**Fig. 2:**
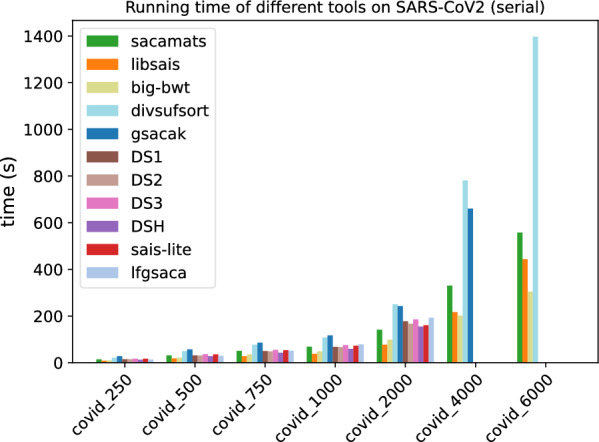
Comparison of running times of different tools (see “Tools compared” section) on subsets of varying length of the SARS-CoV2 dataset (serial implementations)

In Figs. 1 and 2, we display the running time comparison, on both datasets, of our tool and the other seven competitor tools, with full details given in Tables 5 and 6. The grouped bar plot represents a direct comparison of different algorithms on different sizes of input. In the grouped bar plots, whenever there are bars missing, this is because the corresponding tools exceeded the memory limit of 62GB, or, in the case of sais-lite the tool does not support strings longer than $2^{31}$.

**Table 5:** Running times (seconds) for different subset sizes of copies of Chromosome 19 (serial implementations)

Size (MB)	saca- matsmats	lib sais	big -bwt	divsuf sort	gsacak	DS1	DS2	DS3	DSH	sais -lite	lfg saca
250	17.58	9.28	44.70	18.24	31.15	17.94	17.62	19.52	15.11	19.47	15.86
500	31.96	19.16	59.22	39.22	64.53	34.90	33.99	37.60	29.41	39.83	34.09
750	47.38	29.52	70.76	62.14	98.24	52.27	50.76	56.50	44.51	61.85	56.83
1000	62.66	39.49	81.98	85.84	132.11	71.44	69.83	77.28	60.61	82.58	82.75
2000	126.09	81.49	123.63	189.46	278.09	174.05	163.70	182.50	149.36	188.26	200.59
4000	275.53	226.02	205.35	521.91	728.58	474.50	–	–	–	–	–
6000	451.36	404.05	290.41	885.60	–	–	–	–	–	–	–

**Table 6:** Running times (seconds) for different subset sizes of SARS-CoV2 (serial implementations)

Size (MB)	saca- mats	lib sais	big -bwt	divsuf sort	gsacak	DS1	DS2	DS3	DSH	sais -lite	lfg saca
250	14.47	9.09	10.21	21.86	27.79	15.31	15.00	17.37	13.34	17.30	13.85
500	31.48	18.73	21.56	49.18	57.48	31.48	31.00	36.34	27.74	36.02	29.73
750	50.84	28.16	36.28	77.29	85.75	50.12	49.07	55.44	42.80	54.11	51.73
1000	68.62	37.96	48.80	108.29	117.25	67.82	67.17	76.22	59.26	72.76	78.91
2000	141.74	77.06	98.39	250.28	242.97	177.93	167.18	185.61	154.67	161.02	192.63
4000	330.67	216.79	201.92	781.36	660.67	–	–	–	–	–	–
6000	558.13	443.90	304.51	1396.85	–	–	–	–	–	–	–

**Fig. 3:**
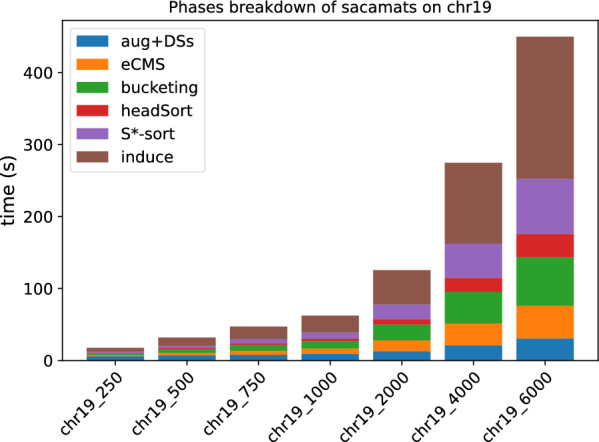
Phases breakdown of sacamats on different subsets of copies of Chromosome 19 (serial implementation)

**Fig. 4:**
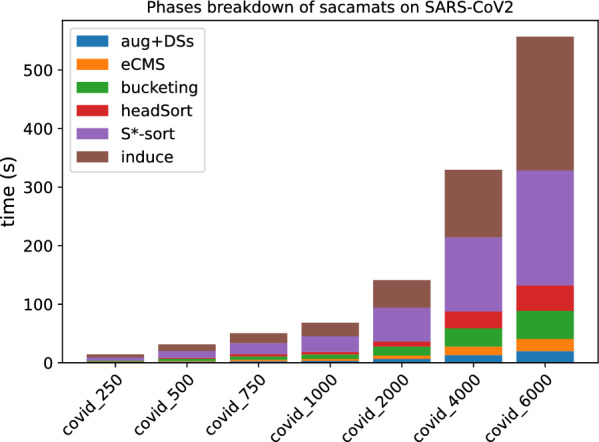
Phases breakdown of sacamats on different subsets of SARS-CoV2 genomes (serial implementation)

**Fig. 5:**
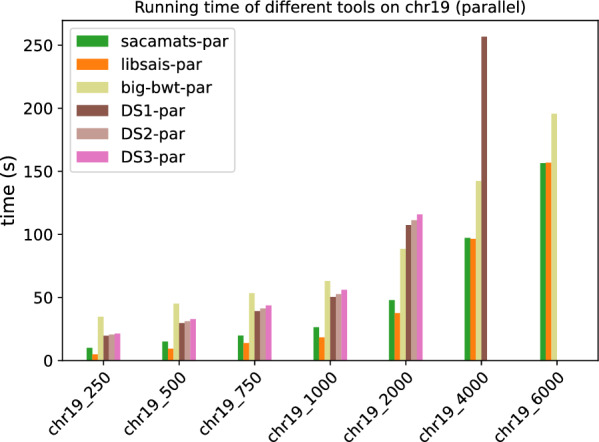
Comparison of running times of different tools (see “Tools compared” section) on subsets of varying length of the Chromosome 19 dataset (parallel implementations)

**Fig. 6:**
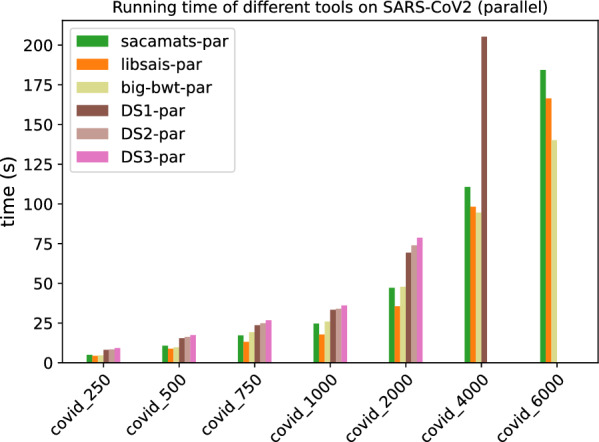
Comparison of running times of different tools (see “Tools compared” section) on subsets of varying length of the SARS-CoV2 dataset (parallel implementations)

**Fig. 7:**
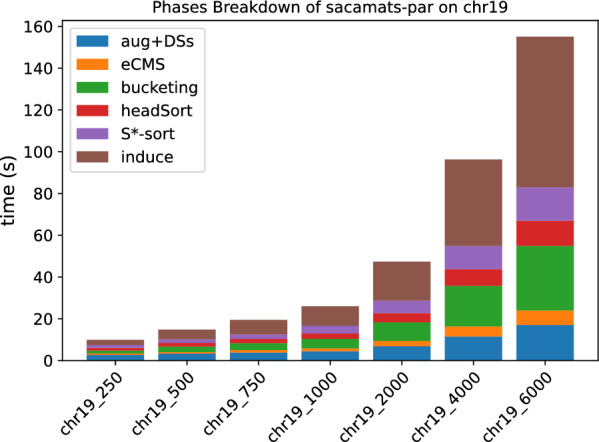
Phases breakdown of sacamats on different subsets of copies of Chromosome 19 (parallel implementation)

**Fig. 8:**
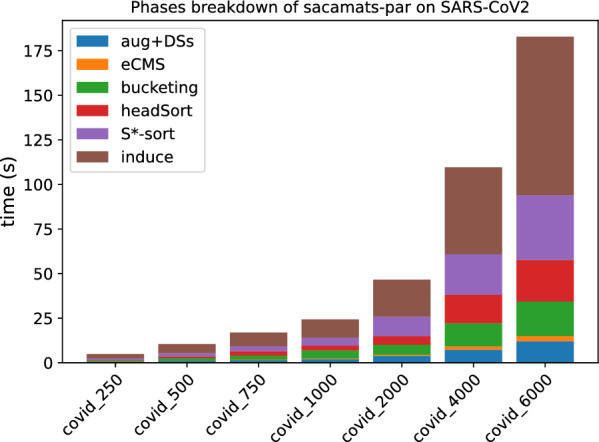
Phases breakdown of sacamats on different subsets of SARS-CoV2 genomes (parallel implementation)

In Figs. 3 and 4, the stacked bar plots show how much each phase of $\texttt{sacamats}$ takes w.r.t. the total running time (cp. “Putting it all together” section). We further show, in Figs. 5 and 6, running time comparisons of parallel implementations, and in Figs. 7 and 8 we have the running time for each phase of sacamats parallel version.

These tools all produce slightly different outputs: divsufsort, sais-lite, gsaca-ds, lfgsaca, and libsais output the $\textit{SA}$, gsacak and sacamats the $\textit{GSA}$, and big-bwt both the $\textit{BWT}$ and the $\textit{SA}$. Because of these differences, if one were to write to disk each result, the running time would be affected accordingly by the size of the output. Therefore, we only compare the building time, i.e. the time spent constructing the SA and storing it in a single array in memory, without the time spent writing it to disk.

#### Running time

By looking at the grouped bar plots (Figs. 1 and 2), one can see that $\texttt{sacamats}$ is competitive on both datasets, in particular, it is faster than all tools on sars-cov2, except big-bwt and libsais. The same is true for chr19, where it is among the fastest methods, and the gain is biggest on larger datasets. Again, the main competitors are big-bwt and libsais.

For example, for the dataset chr19 at 4 GB sacamats takes 276 s. It is faster than gsacak by 164%, divsufsort by 89%, and gsaca-ds (version 1) by 72%. We lose to libsais by 22% and to big-bwt by 34%. On covid dataset at 4GB, sacamats takes 330 s. We are faster than gsacak by 100%, divsufsort by 136%. Again, we lose to libsais by 52%, and to big-bwt by 64%. The results are similar on other dataset sizes, with the gain in time of sacamats over other tools being more pronounced for larger datasets, with the exception of the two tools big-bwt and libsais. This holds for both the covid and chr19 datasets. For full details, see Tables 5 and 6.

Shifting our attention to the stacked bar plots, Fig. 3 indicates that a lot of time is spent in the first phase, consisting in the augmentation of *R* and the construction of various data structures for the augmented version of *R*. In the setting of DNA strings it is not too hard to think that the augmentation process will not elongate *R*, due to the very restricted alphabet. If the application lends itself to it, one could compute beforehand all the data structures listed in Phase 1, gaining roughly between 6 and 30 s of run time, depending on the input size. Alternatively, the common method of replacing N symbols with random nucleotide symbols would be another way to speed up this phase.

##### Parallel implementation comparisons

 In Figs. 5 and 6, we have the running times of tools having a parallel implementation. Every tool was run with a fixed number of threads set to eight.

As one can see, at higher sizes of the Human Chromosome 19 dataset, sacamats is very competitive w.r.t. big-bwt and libsais  outperforming big-bwt at 6GB of data, being 25% faster, and matching libsais. On the SARS-CoV2 dataset, the winner at higher sizes of data is big-bwt, followed by libsais. Our tool is in third place, performing 31% slower than big-bwt and 11% slower than libsais. For full details see Tables 7 and 8.

**Table 7:** Running times (seconds) for different subset sizes of copies of Chromosome 19 (parallel implementations)

Size (MB)	Sacamats-par	Libsais-par	Big-bwt-par	DS1-par	DS2-par	DS3-par
250	10.01	4.80	34.70	19.59	20.54	21.41
500	15.03	9.33	45.14	29.67	31.15	32.78
750	19.72	13.83	53.42	39.20	41.26	43.68
1000	26.32	18.29	63.04	50.38	52.68	56.01
2000	47.85	37.62	88.54	107.34	111.20	115.86
4000	97.22	96.37	142.29	256.72	–	–
6000	156.50	156.80	195.62	–	–	–

**Table 8:** Running times (seconds) for different subset sizes of SARS-CoV2 (parallel implementations)

Size (MB)	Sacamats-par	Libsais-par	Big-bwt-par	DS1-par	DS2-par	DS3-par
250	4.98	4.30	4.66	8.12	8.46	9.25
500	10.72	8.81	9.66	15.42	16.29	17.42
750	17.23	13.19	19.25	23.61	24.85	26.76
1000	24.67	17.77	25.96	33.31	34.03	36.08
2000	47.20	35.52	47.87	69.34	73.92	78.69
4000	110.64	98.22	94.50	205.25	–	–
6000	184.36	166.47	140.14	–	–	–

**Fig. 9:**
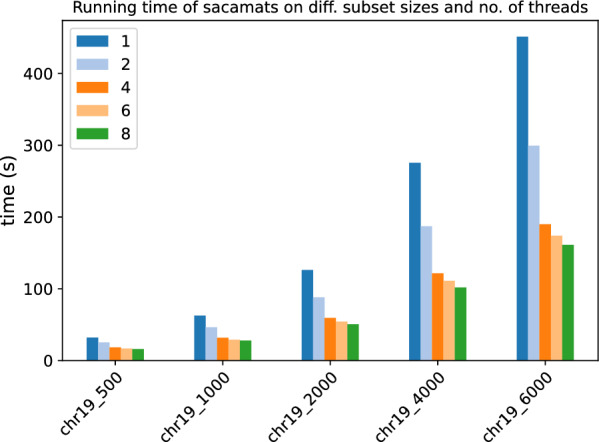
Scaling of our parallel version of sacamats w.r.t. the number of threads used. Here we used different subsets of the Chromosome 19 dataset

**Fig. 10:**
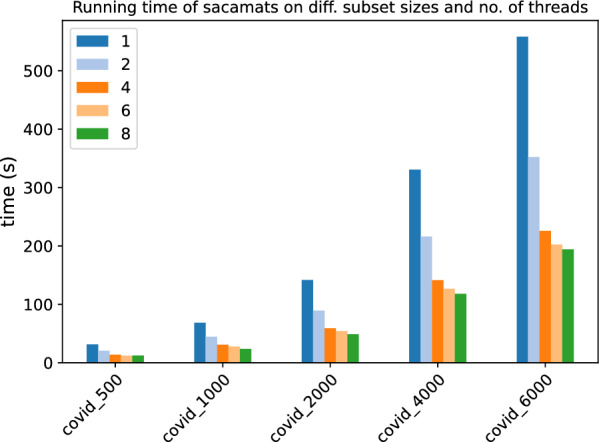
Scaling of our parallel version of sacamats w.r.t. the number of threads used. Here we used different subsets of the SARS-CoV2 dataset

We also show in Figs. 9 and 10 how the running time of our algorithm scales with the number of threads used. It can be seen that using eight threads, our tool takes a third of the time for running on datasets of size 6GB than it does when a single thread of execution is used.

#### Memory consumption

**Fig. 11:**
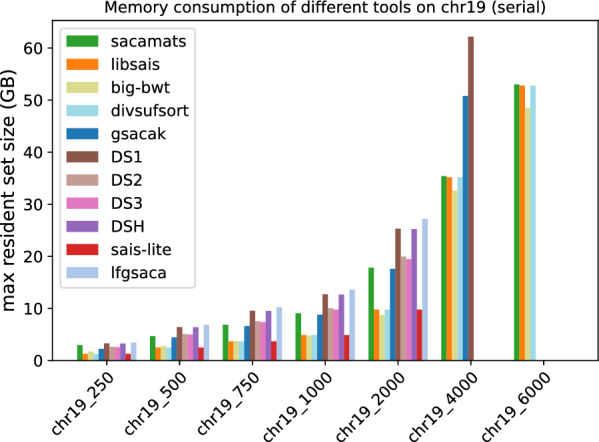
Peak memory measured as maximum resident set size in GB for tools with serial implementation on different subsets of the Chromosome 19 dataset

**Fig. 12:**
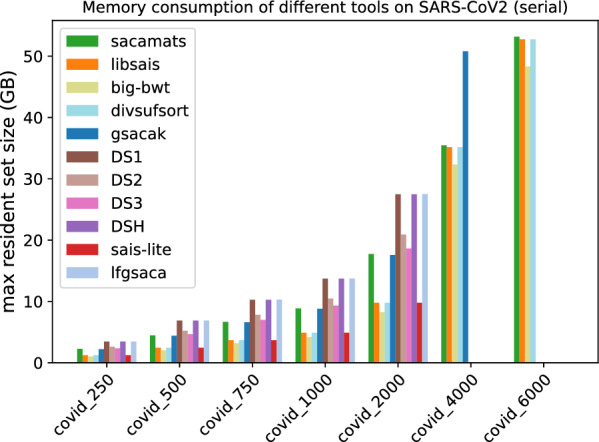
Peak memory measured as maximum resident set size in GB for tools with serial implementation on different subsets of the SARS-CoV2 dataset

Finally, we comment on memory usage (Figs. 11 and 12). We have to make a distinction between sizes of data, because most of the tools use four byte-arrays for sequences up to length $2^{31}$, and then they switch to eight byte-arrays for longer sequences. For the first five datasets, the memory consumption is highest for gsaca-ds and lfgsaca, because they have to keep in memory some extra space for suffix groups. We then have sacamats and gsacak at roughly eight bytes per input symbol, and four bytes per input symbol for divsufsort and sais-lite, libsais, and big-bwt (the $\textit{SA}$ is saved in memory, see in “Tools compared” section). Note that already at these smaller sizes, big-bwt shows the least amount of memory used, due to the fact that the input string is never in memory. On the other hand, big-bwt uses some other internal data structures to build the *SA*. Recall again that we modified the implementation of big-bwt so that it stores the *SA* in memory, instead of streaming it to disk (streaming would reduce memory at the cost of running time).

At 4GB, three out of eight tools run out of memory. For sais-lite  this is because the implementation only handles sequences up to length $2^{31}$ due to the upper limit of four-byte integers.

**Fig. 13:**
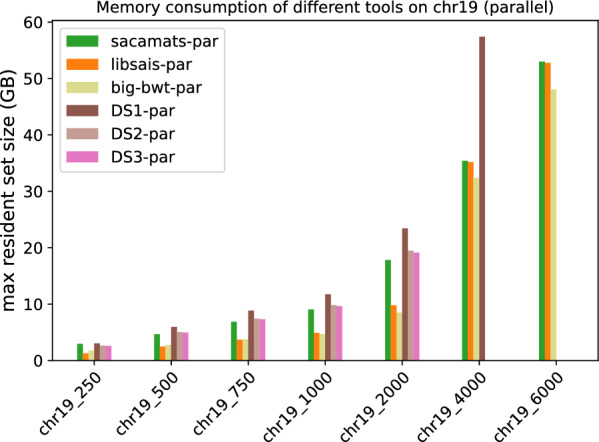
Peak memory measured as maximum resident set size in GB for tools with parallel implementation on different subsets of the Chromosome 19 dataset

**Fig. 14:**
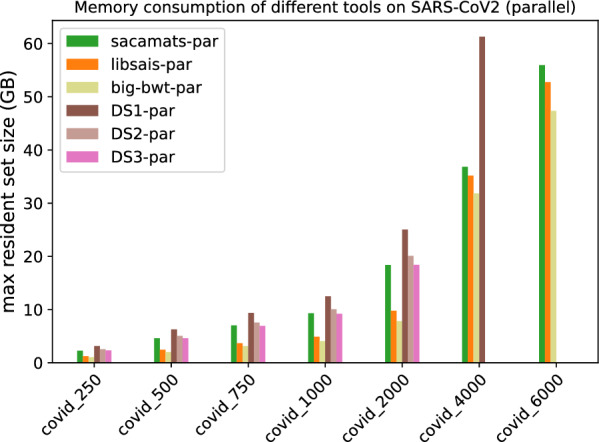
Peak memory measured as maximum resident set size in GB for tools with parallel implementation on different subsets of the SARS-CoV2 dataset

At 6GB, also gsacak runs out of memory. This is because in the implementation it is required to use eight bytes per input character for the *SA* and four bytes per input character for the *DA*. For full details refer to Tables 9 and 10. Similarly, the different parallel versions of gsaca-ds run out of memory at size 6 GB. See Figs. 13 and 14, and Tables 11 and 12 for full details.

**Table 9:** Maximum resident set size (MB) for different subset sizes of copies of Chromosome 19 (serial implementations)

Size (MB)	saca- mats	lib sais	big -bwt	divsuf sort	gsacak	DS1	DS2	DS3	DSH	sais -lite	lfg saca
250	2953	1269	1717	1268	2245	3280	2619	2540	3270	1268	3445
500	4664	2476	2780	2476	4429	6416	5077	4973	6396	2476	6829
750	6878	3684	3775	3683	6613	9558	7546	7379	9528	3683	10214
1000	9062	4891	4782	4891	8797	12,700	10,015	9773	12,661	4891	13,599
2000	17,829	9779	8753	9779	17,591	25,313	19,951	19,456	25,233	9779	27,195
4000	35,400	35,180	32,635	35,180	50,804	62,183	–	–	–	–	–
6000	52,990	52,768	48,494	52,767	–	–	–	–	–	–	–

**Table 10:** Maximum resident set size (MB) for different subset sizes of SARS-CoV2 genomes (serial implementations)

Sze (MB)	saca- mats	lib sais	big -bwt	divsuf sort	gsacak	DS1	DS2	DS3	DSH	sais -lite	lfg saca
250	2258	1224	1007	1224	2200	3455	2622	2334	3455	1224	3444
500	4454	2445	2024	2445	4398	6880	5233	4661	6880	2444	6879
750	6655	3666	3179	3665	6595	10,267	7832	6989	10,267	3665	10,306
1000	8871	4886	4201	4886	8792	13,711	10,449	9319	13,711	4886	13,745
2000	17,739	9769	8252	9769	17,581	27,476	20,909	18,634	27,475	9769	27,499
4000	35,446	35,160	32,335	35,160	50,784	–	–	–	–	–	–
6000	53,162	52,738	48,328	52,738	–	–	–	–	–	–	–

**Table 11:** Maximum resident set size (MB) for different subset sizes of copies of Chromosome 19 (parallel implementations)

Size (MB)	sacamats-par	libsais-par	big-bwt-par	DS1-par	DS2-par	DS3-par
250	2956	1270	1757	3046	2639	2589
500	4669	2478	2758	5945	5034	4961
750	6884	3685	3735	8848	7431	7312
1000	9060	4893	4699	11,751	9827	9653
2000	17,830	9781	8493	23,411	19,465	19,116
4000	35,416	35,185	32,378	57,420	–	–
6000	53,000	52,773	48,049	–	–	–

**Table 12:** Maximum resident set size (MB) for different subset sizes of SARS-CoV2 genomes (parallel implementations)

Size (MB)	Sacamats-par	Libsais-par	Big-bwt-par	DS1-par	DS2-par	DS3-par
250	2262	1226	1057	3155	2531	2315
500	4625	2447	2019	6274	5040	4611
750	7030	3667	3133	9364	7542	6912
1000	9299	4888	4085	12,496	10,056	9211
2000	18379	9771	7846	25,027	20,106	18,400
4000	36,831	35,165	31,828	61,279	–	–
6000	55,950	52,743	47,335	–	–	–

### Effect of repetitiveness on running time

In order to study the role of *eCMS* size, we benchmarked our tool on two sets of simulated data. The two datasets were generated starting from a single reference sequence, a SARS-CoV2 genome in one case, and a single Human Chromosome 19 copy in the other. Starting from this reference, we changed a number of characters in random positions, substituting the DNA character with another one (excluding itself). The number of positions that are changed corresponds to 0.01%, 0.1%, 1%, 5%, respectively 10% of the length of the reference. We concatenated a number of these modified references so that we reach 500 MB of total data.

**Fig. 15:**
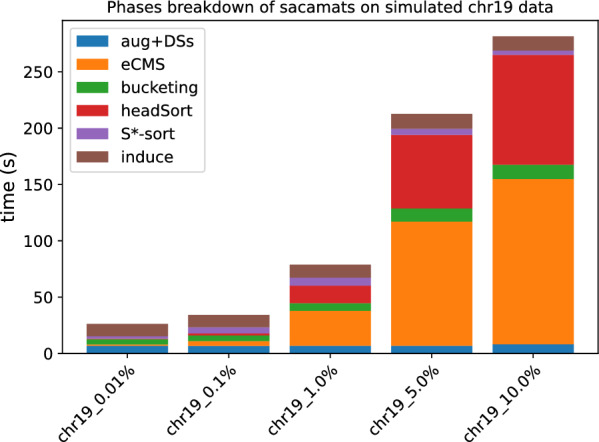
Effect of increasing the number of differences in the sequences of the collection w.r.t. the reference. Here we used simulated Chromosome 19 data. For details see “Effect of repetitiveness on running time” section

**Fig. 16:**
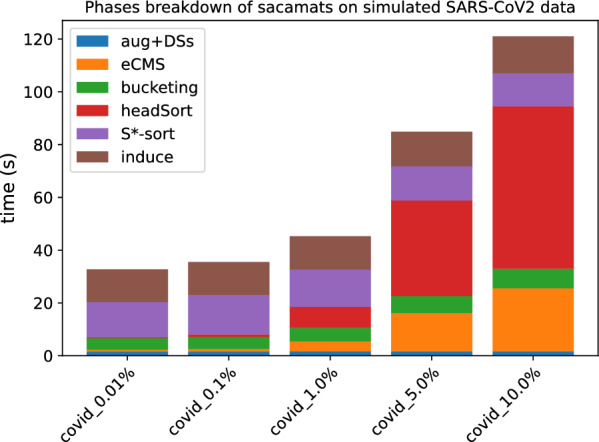
Effect of increasing the number of differences in the sequences of the collection w.r.t. the reference. Here we used simulated SARS-CoV2 data. For details see “Effect of repetitiveness on running time” section

In Figs. 15 and 16, we can see that the total running time starts to increase quite dramatically from 5% of sequence differences. An interesting insight is that only two phases are affected by the increasing number of differences, namely Phase 2 and 4. Phase 2 is impacted by the fact that having shorter matches goes against the heuristics we proposed to speed up the *MS* computation. Phase 4 is instead impacted simply by the number of $\textit{i-head}$s found in Phase 2. The other phases take the same time across different datasets.

## Conclusion

In this paper, we presented a new algorithm for computing the generalized suffix array of a collection of highly similar strings. It is based on a compressed representation of the matching statistics, and on efficient handling of string comparisons. Our experiments show that an implementation of the new algorithm is competitive with the fastest existing suffix array construction algorithms on datasets of highly similar strings, in particular collections of full genome or chromosome sequences.

A byproduct of our suffix sorting algorithm is a heuristic for fast computation of the matching statistics of a collection of highly similar genomes with respect to a reference sequence, which, given the wide use of matching statistics in genomics applications, may be of independent interest. We also envisage uses for our compressed matching statistics (CMS) data structure beyond the present paper, for example as a tool for sparse suffix sorting, or for distributed suffix sorting in which the CMS is distributed to all sorting nodes together with a lexicographic range of the suffixes that each particular node is responsible for sorting. From the CMS alone, each node can extract the positions of its suffixes and then sort them with the aid of the CMS.

Finally, we remark that further optimizations of our tool may be possible. In particular, a semi-external implementation of our approach, in which buckets reside on disk, presents itself as an effective way to reduce main memory usage. In all phases, the actual working set—the amount of data active in main memory—is small (for the most part, proportional to the number of i-heads), and other authors have shown, via highly nontrivial algorithm engineering, that the inducing phase is amenable to external memory, too [38]. We leave these optimizations as future work.

Finally, handling compressed data, such as vcf files, variation graphs [39] or elastic degenerate strings [40, 41], could be beneficial for our algorithm. It is straightforward how to speed up the computation of the *eCMS* data structure in this case. Future research will focus on whether the computation of the *GSA* can also be modified in such a way as to take advantage of the space reduction of compressed input.

## Data Availability

https://github.com/fmasillo/sacamats.

## Footnotes

1 https://www.ncbi.nlm.nih.gov/datasets/coronavirus/genomes/.
